# AKT/PACS2 Participates in Renal Vascular Hyperpermeability by Regulating Endothelial Fatty Acid Oxidation in Diabetic Mice

**DOI:** 10.3389/fphar.2022.876937

**Published:** 2022-07-05

**Authors:** Zhihao Shu, Shuhua Chen, Hong Xiang, Ruoru Wu, Xuewen Wang, Jie Ouyang, Jing Zhang, Huiqin Liu, Alex F. Chen, Hongwei Lu

**Affiliations:** ^1^ Health Management Center, Third Xiangya Hospital of Central South University, Changsha, China; ^2^ Department of Cardiology, Third Xiangya Hospital of Central South University, Changsha, China; ^3^ Department of Biochemistry, School of Life Sciences, Central South University, Changsha, China; ^4^ Center for Experimental Medicine, Third Xiangya Hospital of Central South University, Changsha, China; ^5^ Xiangya School of Medicine, Central South University, Changsha, China; ^6^ Institute for Cardiovascular Development and Regenerative Medicine, Xinhua Hospital Affiliated to Shanghai Jiaotong University School of Medicine, Shanghai, China

**Keywords:** vascular permeability, diabetes, PACS2, fatty acid oxidation, endoplasmic reticulum, mitochondria, endothelial cell

## Abstract

Diabetes is a chronic metabolic disorder that can cause many microvascular and macrovascular complications, including diabetic nephropathy. Endothelial cells exhibit phenotypic and metabolic diversity and are affected by metabolic disorders. Whether changes in endothelial cell metabolism affect vascular endothelial function in diabetic nephropathy remains unclear. In diabetic mice, increased renal microvascular permeability and fibrosis, as well as increased MAMs and PACS2 in renal endothelial cells, were observed. Mice lacking PACS2 improved vascular leakage and glomerulosclerosis under high fat diet. *In vitro*, PACS2 expression, VE-cadherin internalization, fibronectin production, and Smad-2 phosphorylation increased in HUVECs treated with high glucose and palmitic acid (HGHF). Pharmacological inhibition of AKT significantly reduced HGHF-induced upregulation of PACS2 and p-Smad2 expression. Blocking fatty acid β-oxidation (FAO) ameliorated the impaired barrier function mediated by HGHF. Further studies observed that HGHF induced decreased FAO, CPT1α expression, ATP production, and NADPH/NADP^+^ ratio in endothelial cells. However, these changes in fatty acid metabolism were rescued by silencing PACS2. In conclusion, PACS2 participates in renal vascular hyperpermeability and glomerulosclerosis by regulating the FAO of diabetic mice. Targeting PACS2 is potential new strategy for the treatment of diabetic nephropathy.

## Introduction

Diabetic nephropathy (DN) is a chronic microvascular complication of diabetes and has become the leading cause of the end-stage renal disease (ESRD) (Cheng et al., 2021). Endothelial cells are essential participants in the pathogenesis of DN; they constitute the renal filtration barrier (Fu et al., 2019), mediate chronic inflammation (Jeong et al., 2017), and crosstalk with podocytes and mesangial cells (Weil et al., 2012; Kuravi et al., 2014) in progressive DN. Studies have shown that endothelial cell dysfunction, manifested as oxidative imbalance, endothelial apoptosis, abnormal angiogenesis, and decreased nitric oxide bioavailability (McDonald et al., 2019), primarily affects the progression of DN.

According to recent single-cell sequencing, endothelial cells exhibit metabolic transcriptome plasticity in health and disease (Kalucka et al., 2020; Rohlenova et al., 2020). The expression levels of genes related to the fatty acid oxidation (FAO) pathway in resting endothelial cells are 3–4 times higher than those in proliferating endothelial cells (Kalucka et al., 2018). Moreover, inhibiting endothelial glycolysis will reduce the hyperproliferation of tumor blood vessels (Cantelmo et al., 2016). However, the effect of changing endothelial metabolism on diabetes remains unclear. As a systemic metabolic disease, diabetes is often presented with both hyperglycemia and dyslipidemia. It has been reported that nutrient overload can lead to glucolipotoxicity intype 2 diabetes (Dahlby et al., 2020), and hyperglycemia alone is not enough to trigger generalized diabetic microangiopathy (Barrett et al., 2017). Therefore, we speculate that chronic high glucose and high fat (HGHF) conditions may alter endothelial metabolism, leading to diabetic renal vascular dysfunction.

Our previous studies have shown that under high glucose conditions, endothelial mitochondrial DNA damage, increased mitochondrial reactive oxidative species (mtROS) production, and subsequent mitochondrial fission lead to vascular endothelial dysfunction (Liu et al., 2019a; Xiang et al., 2021). Though we and others have explored the pathogenic role of mitochondrial dysfunction in diabetic vascular endothelial cells, the role of endothelial metabolic reprogramming in DN is still unknown. Besides, mitochondria play a central regulatory role in endothelial metabolism, closely related to cell proliferation, apoptosis, and nuclear signal transduction (Qi et al., 2017). In addition, mitochondria-associated ER membranes (MAMs), as a novel subcellular structure, have a more profound impact on endothelial function by regulating mitochondrial dynamics, lipid metabolism, and calcium signaling (Ricciardi and Gnudi, 2020). It has been reported that MAMs have multiple roles in diabetic complications (Wu et al., 2019; Cheng et al., 2020), but no endothelial cells have been concerned. Besides that, papers reported that some MAMs related proteins could play a significant role in diseases. Phosphofurin acidic cluster sorting protein 2(PACS2) (Arruda et al., 2014) regulates cellular energy metabolism in insulin resistance, and fatty acid-CoA ligase 4(FACL4) inhibits the eNOS/NO/cGMP signaling pathway (Yang et al., 2022). Moreover, GRP75 induces mitochondria-associated membrane formation and mitochondrial impairment (Liang et al., 2021). As a cell type with potential metabolic plasticity, hether changes in MAMs are related to endothelial metabolism under diabetic conditions is still worth studying.

In this study, we evaluated the regulation of vascular endothelial metabolism and its role in DN. To this end, we established a streptozotocin (STZ) and high fat diet (HFD)-induced type 2 diabetic mouse model (Wang et al., 2018; Cheng et al., 2019) to evaluate the vascular damage of the kidney. Also, we used an *in vitro* endothelial cell injury model induced by high glucose plus palmitic acid (also referred to as HGHF) (Sun et al., 2021; Wu et al., 2021) to study the main molecular mechanism of barrier function changes and fibrosis.

## Materials and Methods

### Animal Model and Study Design

All animal manipulations were approved by the Institutional Animal Care and Use Committee of Central South University, Changsha, China (#2019sydw0021). All efforts were made to minimize mouse suffering. Eight-week‐old male C57BL/6J mice (wildtype, WT) and phosphofurin acidic cluster sorting protein 2 (PACS2) knockout mice (PACS2^−/−^) were obtained from the Nanjing Biomedical Research Institute of Nanjing University and housed under standard pathogen-free conditions at the Xiangya Medical School of Central South University.

After 1 week of acclimatization, the mice were randomly assigned to the standard chow diet (ND) group and the STZ plus HFD (STZ/HFD) group, with five mice each. The latter was fed HFD (D12109C, Research Diets, New Brunswick, NJ, USA) and intraperitoneally injected 35 mg/kg STZ (S0130, Sigma‐Aldrich, San Louis, MO, USA) twice between 9 and 10 weeks. STZ was dissolved in sodium citrate-hydrochloric acid buffer solution, pH 4.5 (SSC) (Feng et al., 2019; Zhang et al., 2019; Kim et al., 2020). The ND group was injected with the same volume of SSC. Blood glucose was measured at week 11, and mice with blood glucose >11.1 mM were considered to have diabetes. The flow chart of establishing the HFD diabetic mouse model is shown in Supplementary Figure S1A.

### Cell Culture

The results of population studies have shown that patients with diabetes mellitus complicated with hyperlipemia have a higher incidence of rapid progression of diabetic vascular impairment (Haile and Timerga, 2020; Gebreegziabiher et al., 2021). For these patients, glucose control alone cannot reduce the final incidence of ESRD (Coca et al., 2012; Peltier et al., 2014). We believe that glucose and lipid overload are jointly involved in the pathogenesis of high-risk diabetes patients. Therefore, HGHF conditions were created in this study to simulate the disease state (Sun et al., 2021; Wu et al., 2021).

Primary human umbilical vein endothelial cells (HUVECs) were purchased from the American Type Culture Collection (ATCC, Manassas, VA, USA) and cultured in an Endothelial Cell Medium (Invitrogen, Carlsbad, CA, USA) containing 5% fetal bovine serum at 37°C in a 5% CO_2_ incubator. Cells were treated with 30 mM glucose and 0.1 mM palmitic acid (P0500, Sigma-Aldrich, USA) dissolved in 0.5% bovine serum albumin (BSA) for 48 h to simulate HGHF treatment. The Akt inhibitor MK-2206 2HCl was purchased from Selleck Chemicals (S1078, Houston, TX, USA).

### Measurement of Blood Metabolic Parameters

Blood was drawn through the submandibular vein at 11, 14, 17, and 20 weeks to measure serum biochemistry parameters, including total cholesterol (CHO), triglyceride (TG), blood glucose, creatinine (CRE), and blood urea nitrogen (BUN). The 24-h urine microalbuminuria (u-mALB) was measured once a week from the 8th week, but no urine was collected 1 week after each blood collection to avoid the effect of transient blood loss on renal function. All mice were sacrificed at week 20, and kidney tissues were fixed in 4% paraformaldehyde and stained with hematoxylin-eosin (HE) and Masson trichrome.

### SiRNA Transfection

To verify the function of PACS2 in HUVECs, PACS2 siRNA was delivered into cells according to the manufacturer’s instructions. Briefly, cells were transfected with 50 nM PACS2 siRNA or negative control (Ribobio, Guangzhou, Guangdong, China) using Lipofectaminutese 3,000 reagent (Invitrogen) in Opti-MEM I reduced serum medium (ThermoFisher Scientific, Grand Island, NY, USA) for 12 h, and then stimulated with HGHF for another 48 h.

### Masson’s Trichrome Staining

After deparaffinization and rehydration through 100, 95, and 70% ethanol, kidney tissue slides were serially stained in Weigert’s iron hematoxylin working solution for 10 min and Biebrich scarlet-acid fuchsin solution for another 10–15 min. To differentiate nuclei, slides were submerged into the phosphomolybdic-phosphotungstic acid solution for 10–15 min or until the collagen was not red. Slides were then directly transferred into blue aniline solution and stained for 5–10 min. Following differentiation in 1% acetic acid solution for 2–5 min and rapid dehydration through 95% ethyl alcohol and absolute ethyl alcohol, slides were dipped into xylene and finally mounted with a resinous mounting medium.

### Vascular Leakage Assay

As an indicator of vascular permeability, albumin extravasation was evaluated in the Miles assay by measuring the extravasation of albumin-bound Evans blue. In short, mice were anaesthetized with isoflurane inhalation. Five min later, 150 µL of Evans Blue (30 mg/ml; Sigma-Aldrich) was anaesthetized via the tail vein and allowed to circulate for 30 min, then extravasated Evans blue dye was evaluated (Shan et al., 2017). Following photographing the extravasation of Evans blue in the coronal section of kidneys. The kidneys were dried on foil at 150°C for 48 h. To extract Evans blue from the tissue, 500 µL formamides was added and incubated at 55°C for 72 h. Plasma extravasation was quantified by measuring absorbance at 620 nm and calculated per gram of dry weight.

### FITC-Dextran Permeability Assay

Confluent HUVECs were treated with or without HGHF for 48 h on type I collagen-coated culture inserts with 0.4-µm pores (BD Bioscience, Franklin Lakes, NJ, USA). After that, 15 µL of 5 mg/ml FITC-dextran 40 (Sigma-Aldrich) was added to the upper chamber and incubated at 37°C for 15, 30, and 60 min. The fluorescence intensity of FITC-dextran diffused into the lower chamber was measured at Ex/Em = 485/590 nm.

Endothelial cell permeability was detected by using an *In Vitro* Vascular Permeability Assay kit following the manufacturer’s protocol (Millipore, Burlington, MA, USA). After forming a tighter monolayer and exposure to HGHF, the cells were stained with FITC-dextran 40 for 20 min. The amount of FITC-dextran 40 (Aman et al., 2012) diffused across the endothelial monolayer was observed under a fluorescent microscope with a FITC filter and quantified on a fluorescence plate reader.

Leaked ratio = Transwell lower chamber fluorescence intensity (Ex_480_/Em_590_)/upper chamber fluorescence intensity (Ex_480_/Em_590_).

### Oxygen Consumption Assay

The kinetic changes of extracellular oxygen levels, an indicator of aerobic metabolism in living cells, were measured using the MitoXpressXtra oxygen consumption assay kit (Luxel Bioscience, Santa Clara, CA, USA) (Du et al., 2019). Antimycin A, a potent electron transport chain complex III inhibitor, was used to shut down mitochondrial respiration (zero oxygen consumption control). The uncoupling agent carbonyl cyanide 4-(trifluoromethoxy) phenylhydrazone (FCCP) that disrupts the mitochondrial proton gradient was employed to drive its maximal mitochondrial respiration rate. In principle, the phosphorescent signal of MitoXpressXtra is quenched by oxygen and produces a signal that is inversely proportional to the amount of oxygen present. Time-resolved fluorescence (TR-F) was measured at Ex/Em = 380/650 nm and the recommended delay time. The parameters of oxygen consumption included PA-based maximal oxygen consumption rate (PA-OCR) obtained by adding 2.5 μmol/L FCCP plus PA-only-based substrate and negative control (NegOCR) obtained by adding 1 μmol/L antimycin A in the presence of PA-only-based substrate.

### Cellular ATP Assay

Cells were seeded in triplicate in 96-well plates and treated with firefly luciferase for the indicated time. Cellular ATP levels were evaluated using an ATPlite assay (Perkin-Elmer, Waltham, MA, USA) (Liu et al., 2019a).

### NADPH/NADP Measurement

Intracellular NADPH/NADP ratio was assayed using an NADP/NADPH quantification colorimetric kit (K347‐100,BioVision, Milpitas, CA, USA) as per the manufacturer’s instructions.

### Immunohistochemistry

Kidney tissues were fixed in 4% glutaraldehyde at 4°C for 48 h, and then post-fixed in 1% osmium tetroxide at room temperature for 2 h. After pre-staining with barbitone acetate for 10 min, histological samples were dehydrated with acetone and embedded in paraffin wax. Immunohistochemical staining was performed to detect PACS2 (PA5-100167, ThermoFisher) and CD31 (ab28364, Abcam, Cambridge, UK) in endothelial cells and visualized using fluorochrome-conjugated goat anti-mouse (ab150115, Abcam) or anti-rabbit IgG H&L (ab150077, Abcam). PACS2-positive cells were counted and normalized to total endothelial cells in the same field using computer-assisted morphometric analysis.

### Western Blot Analysis

Chemiluminescent based on horseradish peroxidase (HRP) were used to detect and visualize western blots. The following antibodies were used: anti-CPT1α (12252S, Cell Signaling Technology, Beverly, MA, USA), PACS2 (GTX17244, GeneTex, Irvine, CA, USA), VE-cadherin (ab33168, Abcam), β‐actin (A5441, Sigma‐Aldrich), AKT (2938S, Cell Signaling Technology), p-AKT (9018S, Cell Signaling Technology), Smad2 (5339T, Cell Signaling Technology), p-Smad2 (ab280888, Abcam, UK), PPARα (ab215270, Abcam, UK), and HRP conjugated goat anti-rabbit IgG secondary antibody (AS09 602, Agrisera, Vannas, Sweden).

### Electron Microscope

Cells were isolated on nickel fitters, stained with 2% uranyl acetate for 10 min, and then stained with Reynold’s lead citrate for 5 min. The endoplasmic reticulum (ER)-mitochondria contacts of endothelial cells were evaluated by transmission electron microscopy (TEM; Hitachi-7650, Tokyo, Japan) at 60 kV. The ER-mitochondrial contacts were quantified as described previously (Wu et al., 2019). The images were analyzed using ImageJ (National Institutes of Health, Bethesda, MD, USA). The mitochondrial and ER membranes were delineated using the freehand tool. The selected areas were converted to a mask, and the perimeters of ER were calculated. Two independent investigators blindly quantified the images. To quantify MAMs, the total ER perimeter connected to mitochondria to the total ER perimeter was normalized.

### Immunofluorescence Microscopy

HUVECs were labelled with ER-Tracker Blue-White DPX dye (E12353, ThermoFisher Scientific) and Mito Tracker Deep Red FM (M22426, ThermoFisher Scientific) at 37°C for 30 min and observed under a confocal microscope (LSM800, Carl Zeiss Microscopy, Cambridge, MA, USA). The Pearson correlation coefficient mode, a well-defined and generally accepted means of describing the overlap between image pairs, was applied to quantify the degree of co-localization between the fluorophores representing ER-Tracker Blue and Mito Tracker Deep Red. The Pearson correlation coefficient was analyzed using the built-in Carl Zeiss co-localization analysis module from the ZEN software and the threshold obtained from single-label control samples.

### Label-Free Quantitative Proteomics

Total proteins were extracted from HUVECs in the presence or absence of HGHF. Peptide samples (2 μg) were separated and analyzed by Kangchen Biotech (Shanghai, China) using the EASY-nLC1200 system and Q Exactive mass spectrometer (120 min/sample), respectively (Zhu et al., 2020). Differentially expressed peptides were identified at *p* < 0.05 and fold-change > 2, followed by gene ontology (GO) enrichment analysis by using Blast2Go 4.0.7 software.

### Untargeted Metabolomics of HUVECs

Each cell sample is slowly thawed at 4°C, mixed with 1 ml of cold methanol/acetonitrile/H_2_O (2:2:1, v/v/v) and vortexed adequately. The homogenates were sonicated for two cycles at low temperature, 30 min/cycle, incubated at -20°C for 60 min to precipitate proteins and centrifuged at 13,000 rpm at 4°C for 15 min. Supernatants were collected, dried under vacuum, and stored at -80°C. The dried samples were re-dissolved in 100 µL acetonitrile/water (1:1, v/v), vortexed, and centrifuged. The resulting supernatants were used for LC-MS/MS analysis, performed at Applied Protein Technology (Shanghai, China). The metabolites were blasted against the online Kyoto Encyclopedia of Genes and Genomes (KEGG) database (http://geneontology.org/) to retrieve their COs and were subsequently mapped to pathways in KEGG. The corresponding KEGG pathways were finally extracted.

### Statistical Analysis

The SPSS 22.0 software (SPSS, Chicago, IL, USA) was used for statistical analysis. Data are expressed as mean ± standard deviation (SD). Statistical differences were assessed by Student *t*-test of the means between two groups or by one-way analysis of variance of the means between multiple groups. *p* < 0.05 was considered a significant difference.

## Results

### Renal Vascular Hyperpermeability and Glomerulosclerosis in Diabetic Mice

**FIGURE 1:**
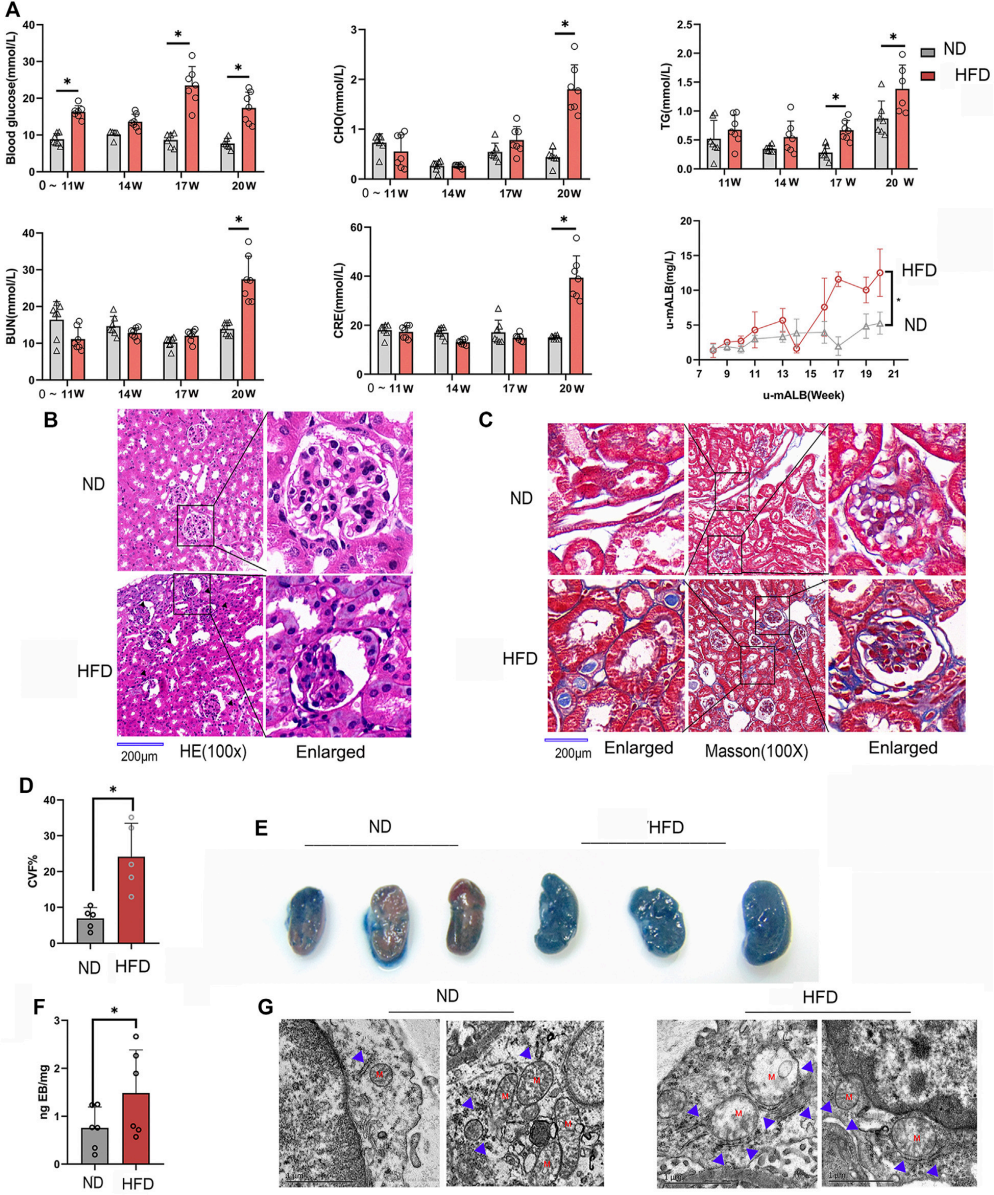
Renal vascular dysfunction and endothelial mitochondria-associated ER membranes (MAMs) increased in the diabetic mice model. **(A)**. Biochemical analysis of blood glucose, total cholesterol (CHO), triglyceride (TG), blood urea nitrogen (BUN), creatinine (CRE), and 24-h urine microalbumin (u-mALB) in mice fed with regular chow diet (ND) or HFD. **(B)**. Hematoxylin-eosin (HE) and **(C)**. Masson’s trichrome staining of mouse kidney tissues. Magnification, ×100 and 400×. Scale bar, 200 µm. **(D)**. Histogram of changes in CVF. **(E)**. Photographs of Evans blue (EB)-stained mouse kidneys. **(F)**. EB extravasation index. Representative images are shown, or data are represented as the mean ± SD, n ≧ 5. **(G)**. Quantitative analysis of mitochondrial MAM coverage in vascular endothelial cells in kidney tissues depicted in Figure 1. Representative images are shown or data are represented as the mean ± SD, *n* = 3. **p < 0.01.

In order to observe the pathological changes of kidney vascular vessels, we created a diabetic mouse model. Hyperglycemia and hyperlipidemia developed after 14 weeks of HFD feeding plus low-doze STZ injection (Figure 1A). Blood CRE, BUN, and u-mALB levels increased significantly at week 20, indicating that the kidney of the HFD group was dysfunctional. Consistent with the serum and urinary biochemical analysis results, HE and Masson trichrome staining showed pathological changes in renal tissues, manifested as atrophy of glomerular vascular loops (Figure 1B) and renal microvascular fibrosis (Figures 1C,D). Moreover, Evans blue extravasation demonstrated an increased renal tissue extravasation index in the HFD group (Figures 1E,F). TEM observation of renal endothelial cells showed a significant increase in MAMs of renal cortical glomerular endothelial cells, accompanied by mitochondrial swelling and crista degeneration (Figure 1G). These results indicate that mitochondria are involved in endothelial cell dysfunction induced by HGHF.

### Increased MAMs and Barrier Dysfunction in HGHF-Treated Endothelial Cells

**FIGURE 2:**
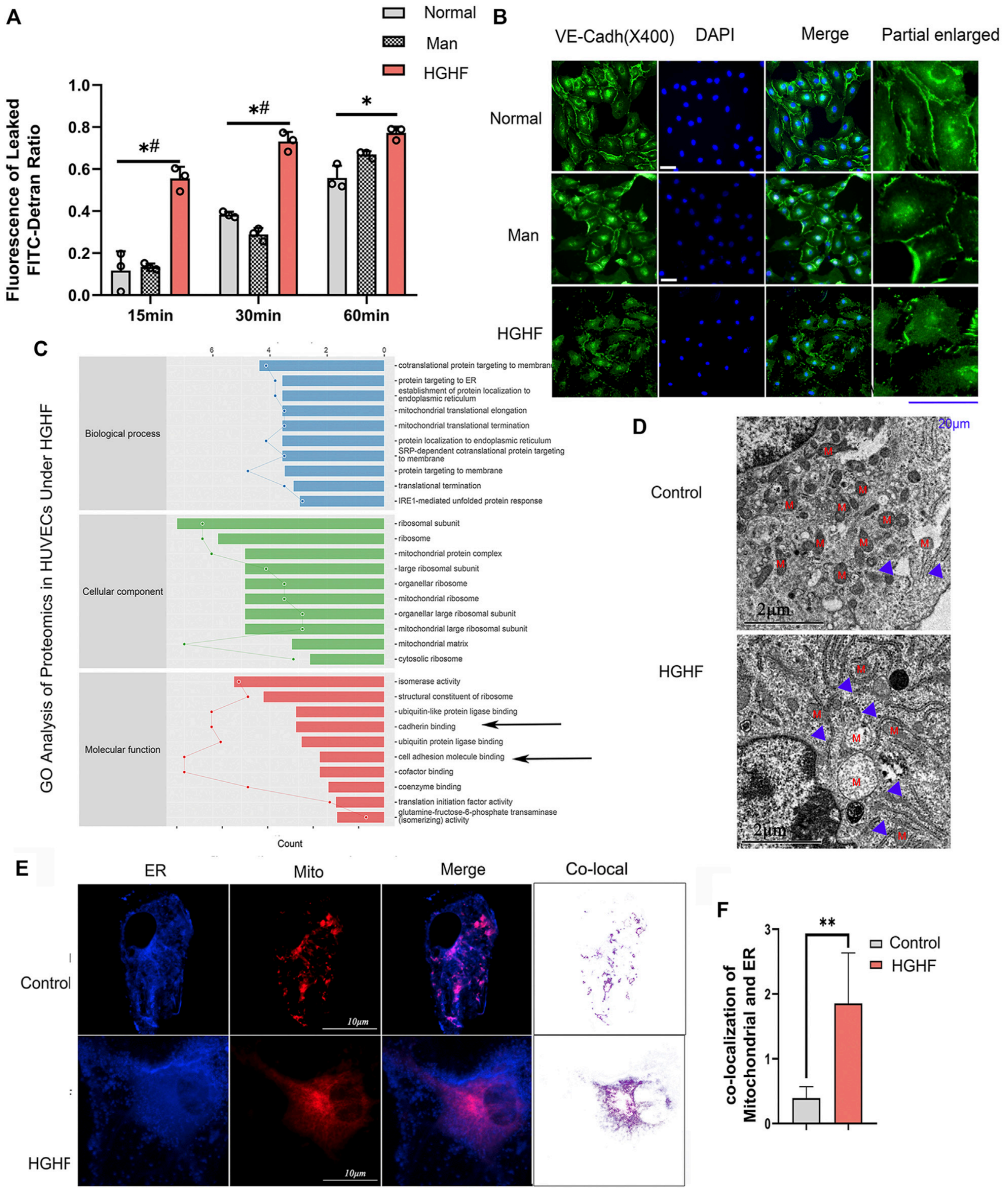
Effect of HGHF on endothelial barrier function and MAMs in endothelial cells. **(A)**. Cells were treated with 30 mM glucose and 0.1 mM palmitic acid (HGHF) for 48 h, FITC-dextran leakage ratio was quantified at 15, 30, and 60 min after adding the dye. Normal, 0.5% bovine serum albumin (BSA); Man, 30 mM mannitol in 0.5% BSA. Data are represented as the mean ± SD, *n* = 3. *p < 0.05 for HGHF *vs*. Normal, #*p* < 0 .05 for HGHF *vs*. Man. **(B)**. Representative images of immunofluorescence staining of VE-cadherin (VE-cadh, green) and DAPI (blue). Scale bar, 20 µm. **(C)**. GO ontology analysis of changed cellular components, biological processes, and molecular functions in HUVECs treated with HGHF. **(D)**. Transmission electron microscope imaging of HUVECs Blue arrow: MAMs. M: mitochondria. **(E)**. Confocal imaging and Transmission electron microscope imaging of HUVECs double-labeled with ER-Tracker Green and Mito Tracker Deep Red. Bar, 5 µm. **(F)**. Quantitative analysis of the co-localization fluorescence intensity of mitochondria and ER in B.

To explore the underlying mechanism behind endothelial cell dysfunction in diabetic mice, we next examined the barrier function changes of endothelial cells. As shown in Figure 2A, FITC-dextran permeability was significantly increased in the HGHF group from 15, 30 until 60 min. VE-cadherin is the central adhesion molecule of endothelial cell-cell junctions (Lampugnani et al., 2018). The dissociation of p120-catenin and β-catenin from VE-cadherin complexes will increase VE-cadherin internalization and weaken the endothelial barrier (Yang et al., 2015; Juettner et al., 2019). The immunofluorescence of VE-cadherin showed a significant decrease from the cell membrane in Figure 2B, indicating that the adherent contact between endothelial cells was damaged.

By mining the MS reference database, protein identification was performed on data-independent acquisition (DIA) data results (identification criteria: precursor threshold, 1.0% false discovery rate (FDR) and protein threshold, 1.0% FDR). We identified 26,201 peptides and 3,678 proteins in the two sets of samples. The identified proteins were screened for differentially expressed ones between the ND and HGHF groups. The relative fold change of expression >1.2 and the Student *t*-test q-value < 0.05 were used as filter criteria to process DIA quantitative data. On this basis, 320 differentially expressed proteins were selected out (Supplementary Figure S2A). Of them, 138 were upregulated and 182 downregulated after HGHF treatment. GO annotation analysis showed that they were mainly enriched in the unfolded protein response in the ER, and the mitochondrial translational elongation and termination processes (Figure 2C) indicate mitochondrial damage or ER stress. Proteomics also supported that the proteins were enriched in the mitochondria and ER (Supplementary Figure S2B). Several proteins related to renal damage and inflammation increased, consistent with the chronic renal inflammation state (Supplementary Figure S2C). We also found that the expression of the integrin-related proteins (like ITGB4, ITGA3) and cell-cell adhesions protein MLLT4 had down-regulated significantly (Figure 2C), which indicates damage to membrane integrity (Tremblay et al., 2014). Further, we verified the potential changes in mitochondria-ER contact *in vivo*. The same cell samples were observed under TEM, showing enrichment of ER and MAMs (Figure 2D) under HGHF. Double staining of mitochondria and ER revealed their more significant co-localization in HGHF-treated HUVECs (Figures 2E,F).

### Increased Expression of PACS2 in HGHF-Treated Endothelial Cells

**FIGURE 3:**
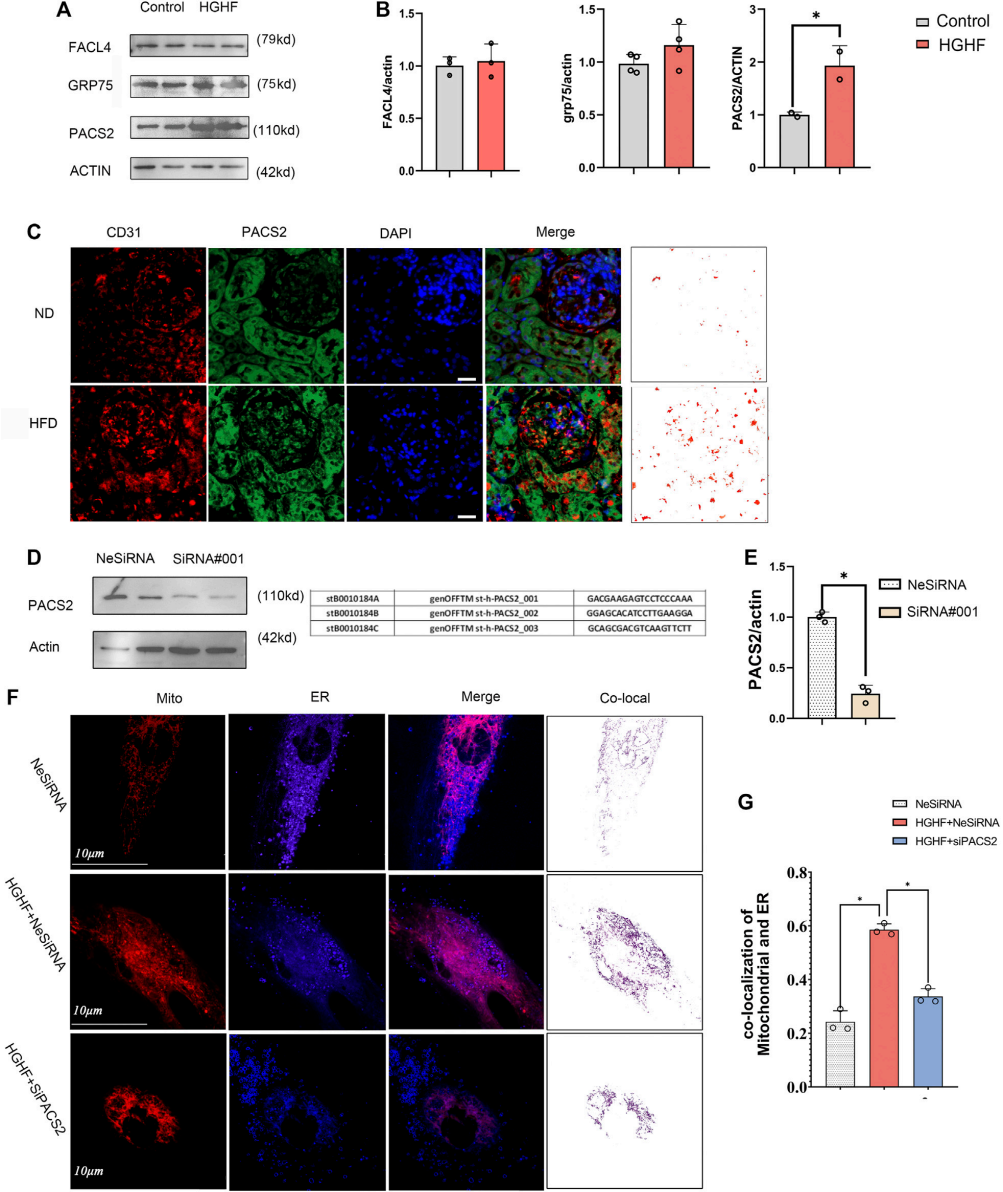
PACS2 increased expression in endothelial cells response to HGHF at the MAMs. (A). Western blot analysis of MAM-related proteins. ACTIN used as loading control. **(B)**. Quantitative analysis of the expression of FACL4, GRP75, and PACS2 in A. The band densities were quantitated and normalized to the corresponding ACTIN. **(C)**. Immunofluorescence imaging of cells stained for CD31 (red), PACS2 (green), and nucleus (DAPI, blue) in mouse kidney tissues. **(D)**. Transfection efficiency of PACS2 siRNA in HUVECs. **(E)**. Quantitative analysis of PACS2 knockdown in D. **(F)**. Confocal imaging of cells double-labeled with ER-Tracker Blue and Mito Tracker Deep Red in HUVECs. Single optical sections are shown and the purple ones indicate the co-localization of mitochondria and ER. Bar, 10 µm. **(G)**. Quantitative analysis of the co-localization ratio in F. Representative images are shown, or data are represented as the mean ± SD, *n* = 3. *p < 0.05.

Based on the TEM and immunofluorescence observations of renal endothelial cells *in vivo* and HGHF-treated HUVECs *in vitro*, we further explored the expression changes of three MAMs regulatory proteins, acyl-CoA synthetase 4 (FACL4) (Lewin et al., 2002), PACS2 (Yang et al., 2021), and glucose-regulated protein 75 (GRP75) (Liu et al., 2019b), in HUVECs under HGHF exposure. Among these regulatory proteins, PACS2 was significantly increased in HGHF-induced HUVECs (Figures 3A,B), and it also had an inevitable increase in diabetic mouse renal vascular tissues (Figure 3C). To further understand the role of PACS2, we silenced PACS2 in HUVECs using siRNA (Figures 3D,E). We found knocking down PACS2 could attenuate HGHF-enhanced co-localization of mitochondria and ER (Figures 3F,G). These results indicate that PACS2 is an active regulator of MAMs and may be involved in endothelial cell dysfunction in HGHF-treated endothelial cells.

### Loss of PACS2 Expression Improves HGHF-Induced Endothelial Barrier Dysfunction

**FIGURE 4:**
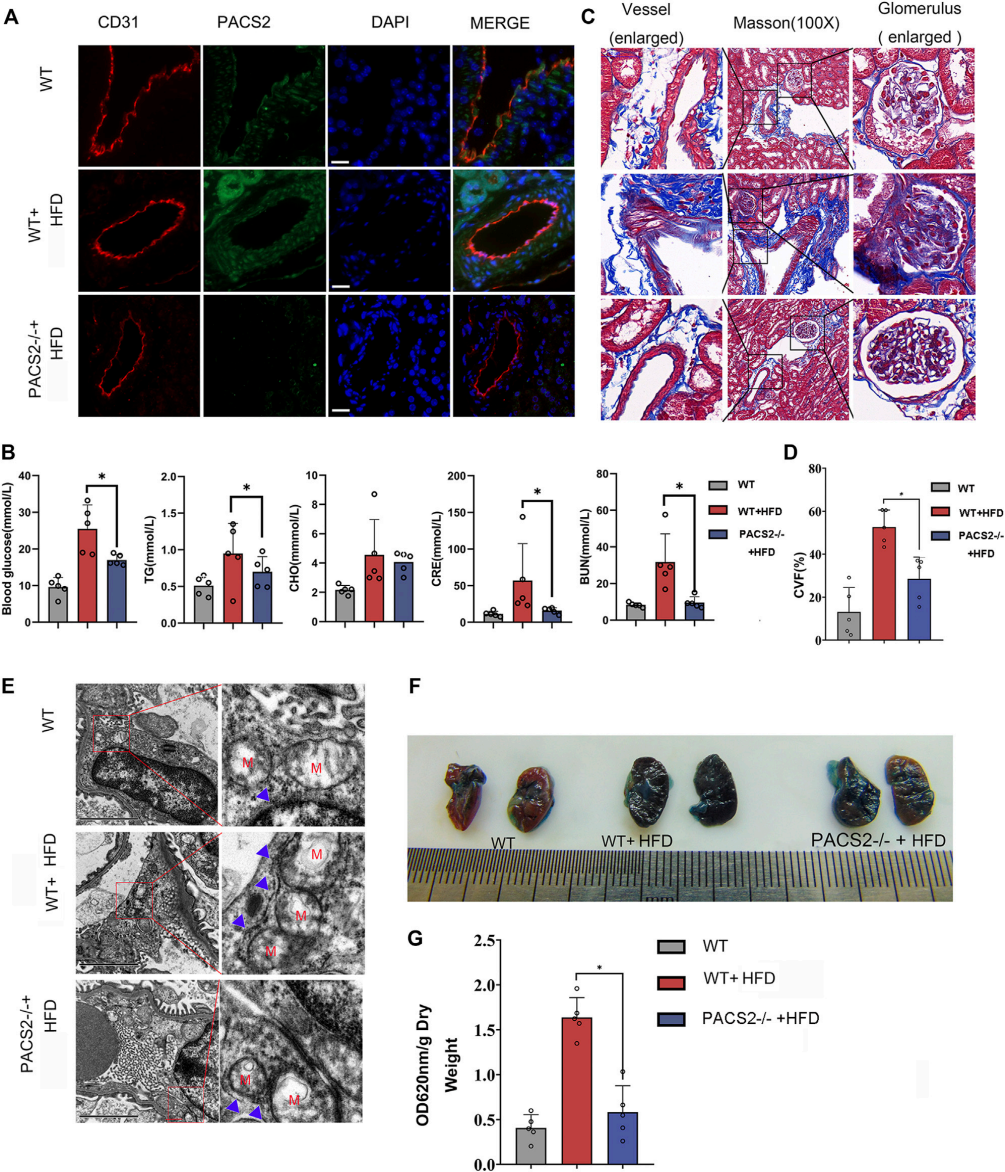
Loss of PACS2 expression improves kidney barrier function and anti-fibrosis induced by HGHF. **(A)**. Immunofluorescence of kidney tissues. Bar, 50 μm **(B)**. Biochemical analysis of serum parameters in wild type (WT) and PACS2^−/−^ mice fed with or without HFD. **(C)**. Masson staining of kidney tissues, Magnification, ×100. **(D)**. Histogram of CVF in B. **(E)**. Transmission electron microscope imaging of mouse kidney ultrastructure. Magnification, ×15,000. Red M, mitochondria of renal endothelial cells; blue arrow, MAMs. Images of Evans blue staining of mouse kidneys. **(F)**. Photographs of Evans blue (EB)-stained mouse kidneys. **(G)**. Histogram of EB extravasation index. Representative images are shown or data are represented as the mean ± SD, n ≥ 5. *p < 0.05.

PACS2 knockout mice (PACS2^−/−^) were used to confirm the effect of PACS2 on vascular endothelium under HGHF *in vivo*. Diabetic PACS2^−/−^ mouse modeling was created the same as depicted in Supplementary Figure S1. Microscopic immunofluorescence observations verified the gene knockout efficiency (Figure 4A). We observed that the HFD treatment altered the biochemical parameters of renal function in WT mice. However, PACS2 knockout eliminated the increase in blood glucose, TG, CRE, and BUN caused by HFD treatment. However, it did not affect CHO (Figure 4B), indicating that PACS2 is implicated in HFD-induced renal dysfunction.

Compared to HFD-treated WT mice, PACS2-deficient mice showed a significant reduction in HFD-induced glomerular fibrosis in Masson staining (Figures 4C,D). In addition, we found that the glomerular cortex endothelial cells and vascular endothelial cell MAMs in the kidney tissues of PACS2^−/−^ + HFD mice were significantly reduced (Figure 4E), as was the glomerular Evans blue leakage index (Figures 4F,G), suggesting that PACS2 plays a role in HFD-induced kidney injury by regulating MAMs.

### Inhibition of AKT Blocks Hyperpermeability and Fibronectin Generation Under HGHF With PACS2 Upregulation

In order to explore how HGHF-induced endothelial PACS2 and MAMs vary, the upstream regulators of PACS2 were then examined. Recent reports noted that AKT could mediate vascular barrier leakage and inhibit renal fibrosis (Lewin et al., 2002; Yang et al., 2021) and controlling MAMs integrity (Peltier et al., 2014). Therefore, we evaluated whether AKT works upstream of PACS2 in HUVECs treated with HGHF.

**FIGURE 5:**
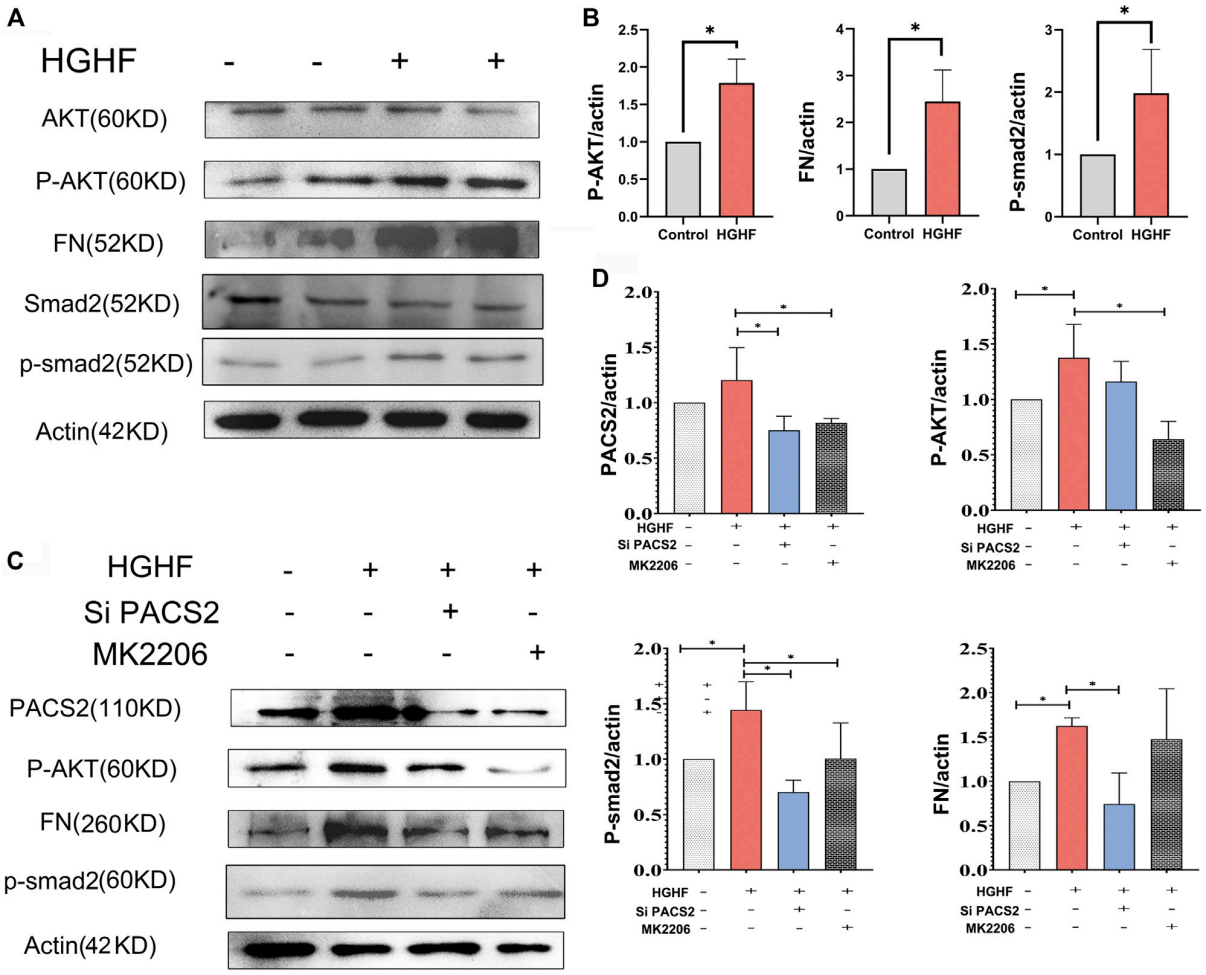
PACS2 regulates barrier injury and p-Smad2 under HGHF blocked by inhibition of the p-AKT pathway in HUVECs. **(A)**. Western blot analysis of FN, p-smad2 and p-AKT expression under HGHF. **(B)**. Quantitation and normalization of FN, p-smad2 and p-AKT expression in A. **(C)**. Western blot analysis of PACS2, FN and p-smad2 and p-AKT expression under p-AKT inhibitor MK2206 and siPACS2. **(D)**. Quantitation and normalization of proteins expression in C.

Figures 5A,B show that HGHF induced endothelial cell fibrosis, indicated by increased FN expression. At the same time, the phosphorylation levels of AKT and Smad2 were significantly increased (Figures 5A,B), suggesting that HGHF activates both AKT and Smad2 signaling pathways. Extracellular matrix (ECM) is secretable from endothelial cells, and its accumulation is the pathological basis of DN. However, the transcription factor Smad2 signaling mediates endothelial-related ECM deposition and renal fibrosis (Weng et al., 2020). To understand the potential role of PACS2 in the activation of AKT and Smad2, we silenced PACS2 and then treated cells with HGHF. We found that knocking down PACS2 reduced FN expression and Smad2 phosphorylation but did not inhibit AKT phosphorylation, indicating that PACS2 regulates Smad2 signaling during HGHF-induced endothelial cell fibrosis. Interestingly, MK2206, a selective AKT inhibitor, decreased HGHF-induced PACS2 expression and Smad2 phosphorylation, indicating that AKT signaling works upstream of PACS2 regulation under HGHF treatment (Figures 5C,D). These results indicate that HGHF-induced PACS2 upregulation in endothelial cell dysfunction is partly through the AKT signaling.

### Inhibition of AKT/PACS2 Improves Free Fatty Metabolism in HGHF-Treated HUVECs

**FIGURE 6:**
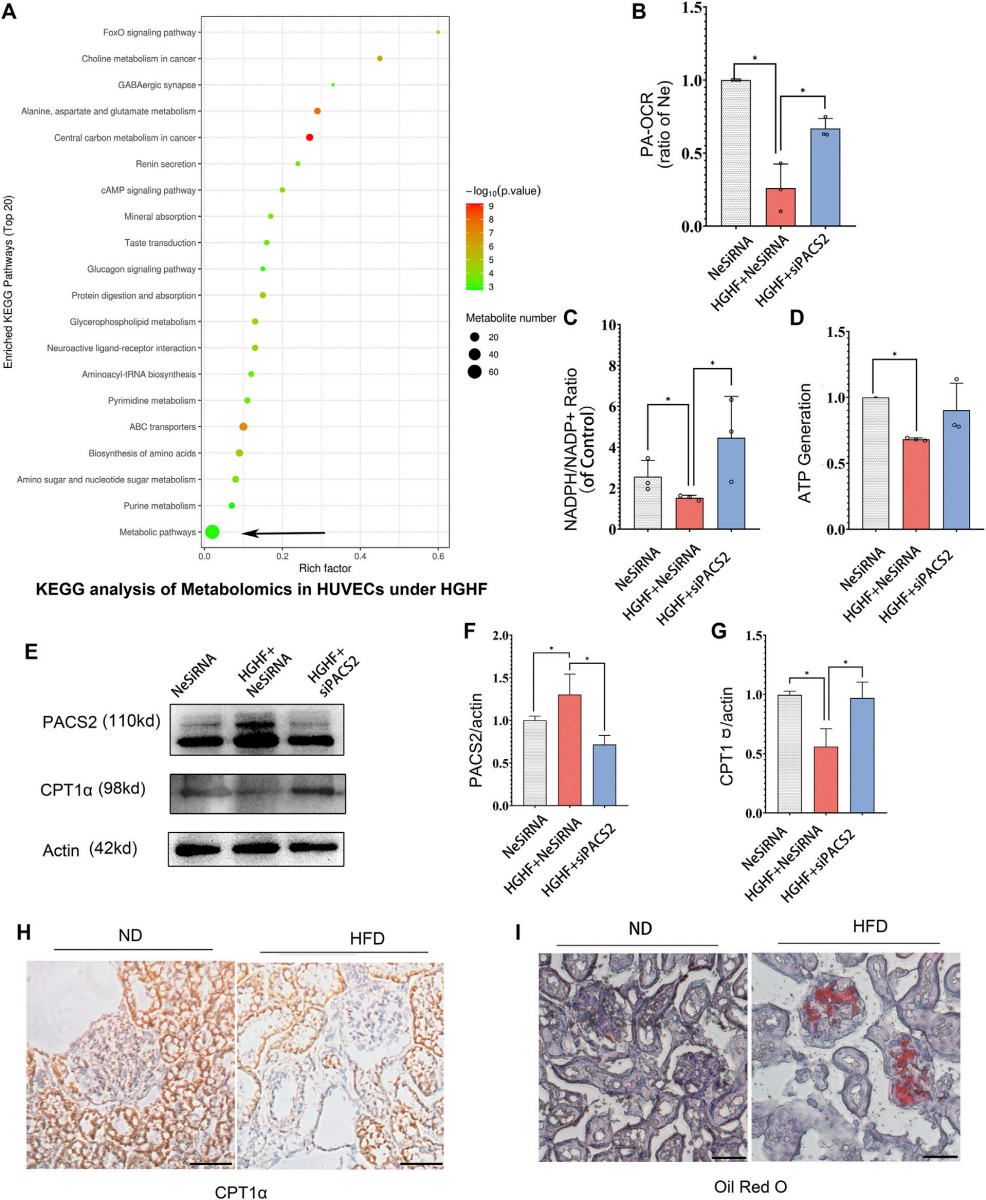
Silencing PACS2 improves free fatty metabolism in HUVECs treated with HGHF. **(A)**. KEGG analysis of metabolomics in HUVECs under HGHF. **(B)**. Palmitic acid (PA)-based oxygen consumption rate (OCR) was measured in cells transfected with negative siRNA (NeSiRNA) or PACS2 siRNA (siPACS2) and treated with or without HGHF. **(C)**. Determination of cellular NADPH/NADP ratio. **(D)**. Determination of cellular ATP levels. **(E)**. Western blot analysis of PACS2 and carnitine palmitoyl-transferase 1α (CPT1α) expression. **(F)**. Quantitation and normalization of PACS2 expression in E. **(G)**. Quantitation and normalization of CPT1α expression in E. **(H)**. Immunohistochemical staining of CPT1α in mouse kidney. Magnification, ×200. **(I)**. Oil Red staining of mouse kidney. Magnification, ×200. Representative images are shown or data are represented as the mean ± SD, *n* = 3. *p < 0.05.

Recent research reported that PACS2 could regulate cellular energy metabolism, in which the free fatty metabolism was also related to cell-cell junction (Xiong et al., 2018). In order to understand how AKT/PACS2 signaling impacts endothelial barrier function, we explored the metabolic index in endothelial cells. Non-targeted metabolomics were tested under HGHF induced HUVECs and FAO’s key enzyme, carnitine palmitoyl-transferase 1α (CPT1α), was examined in diabetic kidney tissue. KEGG pathway analysis results revealed that most differential metabolites were enriched in the metabolism pathways (Figure 6A). Significant changes were found in adenosine monophosphate, adenosine 5′-diphosphate, glycerol 3-phosphate (phosphoglycerol 3), N-acetylglucosamine-1-phosphate, and phosphorylcholine (Supplementary Figure S2), all of which are related to fatty acid metabolism, indicating that fatty acid metabolism is easily affected by HGHF and has a role in HGHF-induced HUVEC damage.

Next, we measured FAO ability through mitochondrial respiration using PA as an energy substrate. We evaluated FAO-dependent OCR (PA-OCR) by adding FCCP or etomoxir (ETO) into cultured HUVECs. The results showed that PA-OCR was significantly reduced after HGHF exposure. However, silencing PACS2 reversed the inhibition of PA-OCR by HGHF (Figure 6B). Quantitative NADPH/NADP^+^ (Figure 6C) and ATP (Figure 6D) analyses showed that the NADPH/NADP^+^ ratio and ATP production in HUVECs was significantly reduced after HGHF treatment. However, knocking down PACS2 prevented the effect of HGHF on the NADPH/NADP^+^ ratio and showed a tendency to block the effect of HGHF on ATP production. These results suggest that HGHF interferes with mitochondrial aerobic respiration, where PACS2 is a positive regulator.

We also found that, contrary to the changes in PACS2 expression, CPT1α expression was significantly reduced under HGHF stress but was rescued by PACS2 knockdown (Figures 6E–G). This result was consistent with the CPT1α expression in kidney tissues of diabetic mice fed with HFD and found that its protein expression was reduced (Figure 6H) in Oil red-positive tissues (Figure 6I). These results further suggest that PACS2 regulates fatty acid metabolism, possibly by disturbing the balance of endothelial redox homeostasis through NADPH production.

### Inhibition of FAO Blocks PACS2 Regulation of Endothelial Barrier Function and Hyperpermeability

**FIGURE 7:**
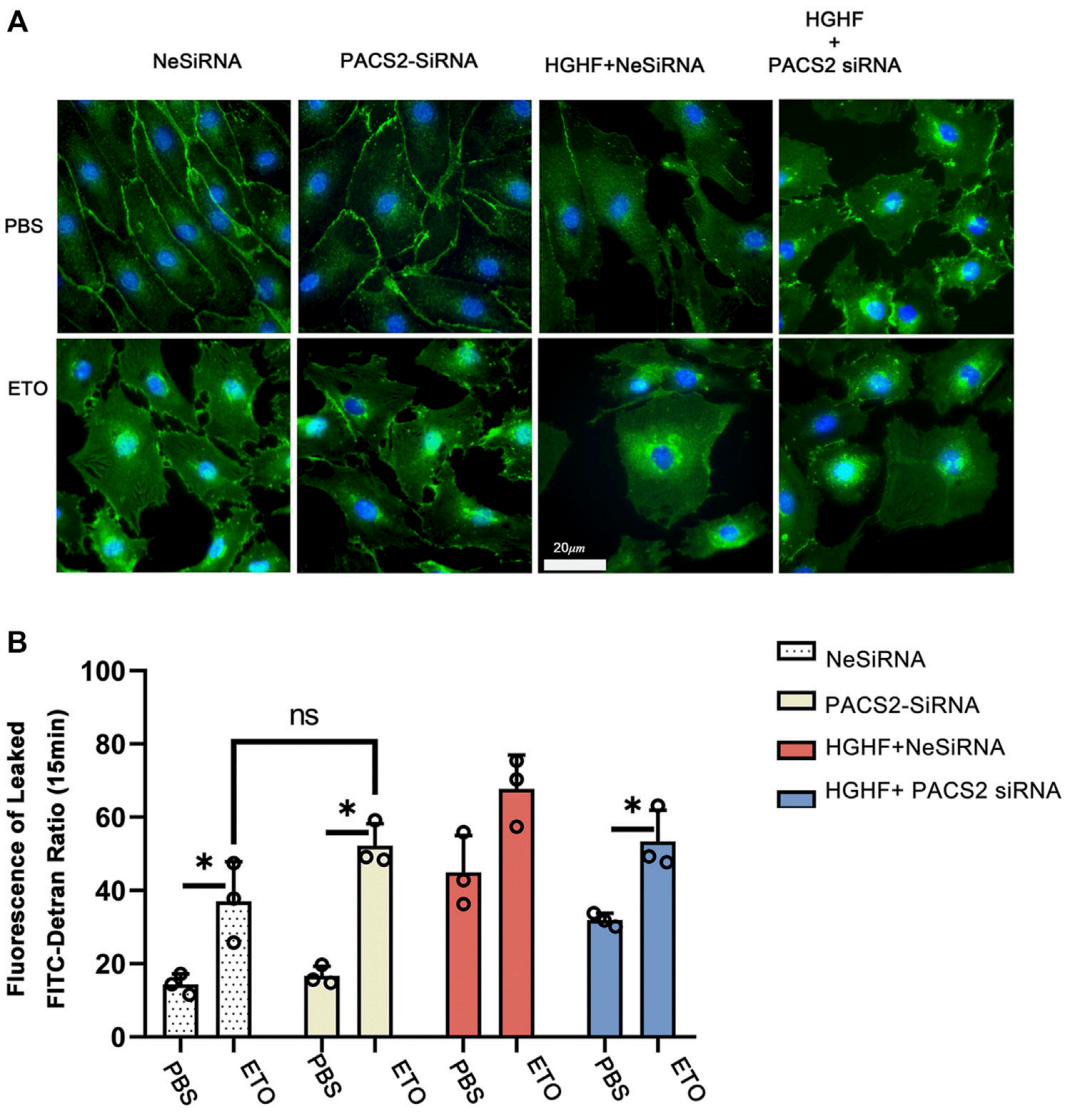
Inhibition of fatty acid β-oxidation disturbs the barrier function of endothelial cells and eliminates the protective effect of PACS2. **(A)**. Immunofluorescence staining of VE-cadherin (green) in cells transfected with NeSiRNA or siPACS2 and treated with the fatty acid β-oxidation inhibitor etomoxir (ETO) or phosphate buffered saline (PBS) as vehicle control. Blue, DAPI. Bar, 20 µm. **(B).** Quantification of FITC-dextran leakage ratio of HUVECs within 15 min. Representative images are shown or data are represented as the mean ± SD, *n* = 3. *p < 0.05.

As observed above, HGHF affects endothelial FAO. However, it is unclear if PACS2 regulation of barrier function under HGHF is reliable for the FAO change. Therefore, the FAO inhibitor ETO were used to block FAO in endothelial cells to test this. We found that ETO increased VE-cadherin internalization (Figure 7A) and FITC leakage (Figure 7B) under non-HGHF conditions. Moreover, PACS2 knockdown attenuated the effect of HGHF on FITC leakage and VE-cadherin internalization (Figure 7B). However, this PACS2 effect was not seen when ETO was present. These results indicate that FAO is downstream of PACS2 in regulating endothelial barrier function under HGHF.

## Discussion

**FIGURE 8:**
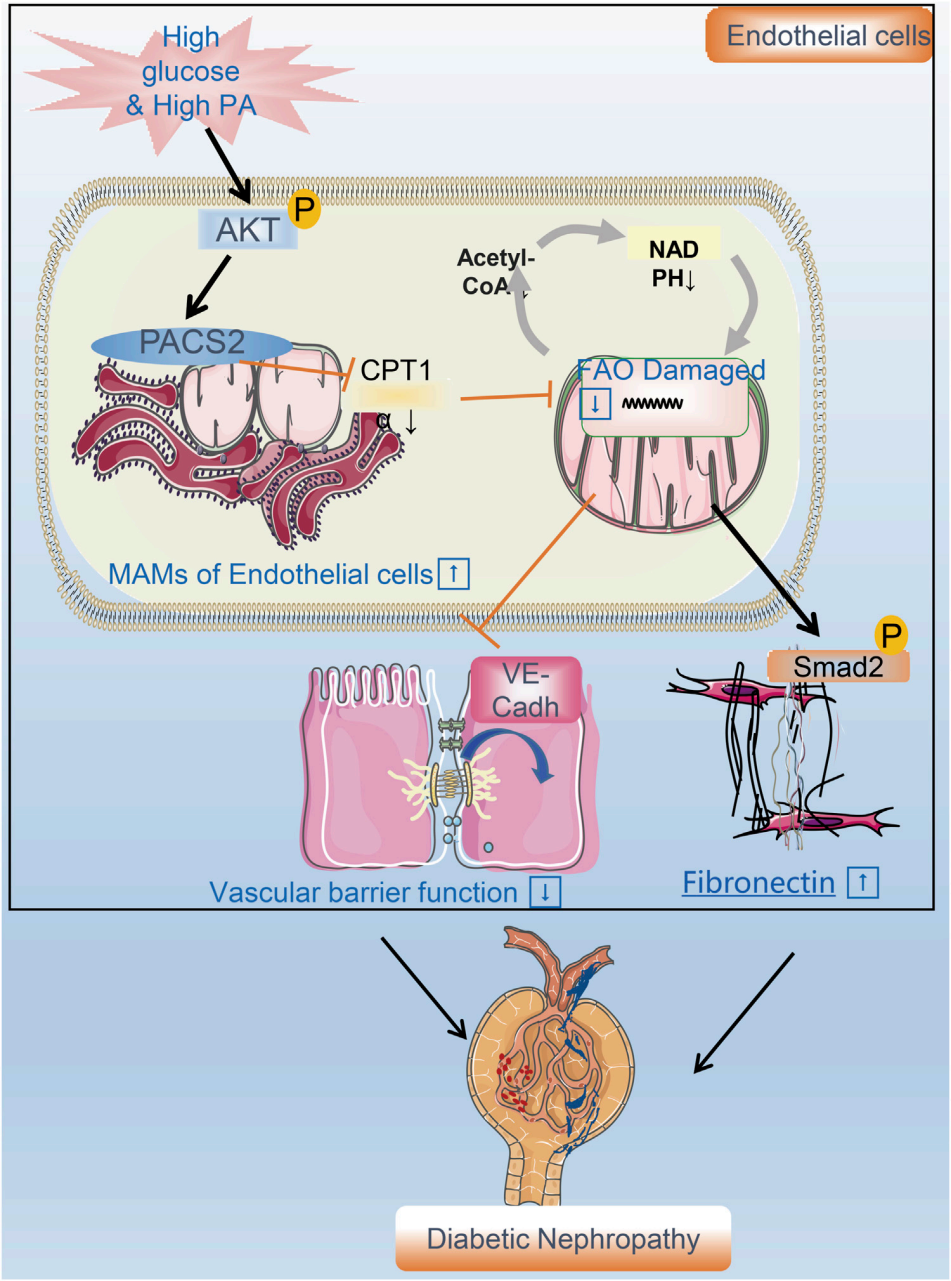
A flow chart model proposal to explain the mechanism of renal vascular endothelial cell injury in DN. PACS2 is a critical component of the mechanism behind DN caused by HGHF. Under high glucose/high fat conditions, PACS2 is upregulated and increases the number of MAMs, followed by a decrease in CPT1α expression, free fatty (FA) β-oxidation, and NADPH production in renal vascular endothelial cells. All these metabolic changes will promote the internalization of VE-cadherin, disturb the barrier function of endothelial cells, and fibrosis increased ultimately lead to pathogenesis and development of DN.

Diabetes combined with hyperlipidemia is usually regarded as a potent risk factor to induce DN. Endothelial cells are the fundamental component of the renal filtration barrier and are directly damaged by abnormal circulating metabolites in diabetes. This study aimed to determine the function of vascular endothelial cells and their metabolic mechanisms in the occurrence and development of DN. We found that the expression of the endothelial MAM regulatory protein PACS2 increases significantly in response to HGHF. Furthermore, the knockout of PACS2 protects renal vascular function from HGHF. Moreover, PACS2 regulates endothelial FAO and further affects VE-cadherin internalization and Smad2 activation, which damage the filtration membrane barrier and increase FN generation in the ECM. (Figure 8).

Due to the low content of mitochondria (2–6% of the cytoplasm volume), endothelial cells preferentially obtain 80% of ATP through glycolysis in a resting state (Caja and Enríquez, 2017). Although the energy supply from FAO is limited, it is essential for endothelial redox homeostasis (Kalucka et al., 2018). It was reported that when assembled into a formed network, HUVECs will increase FAO, decrease glycolysis, and increase NADPH regeneration to maintain redox homeostasis (Patella et al., 2015; Andrade et al., 2021). However, when both glucose and lipids are overloaded, malonyl-CoA will accumulate and interact with CPT1α, thereby reducing the oxidation of fatty acids in the mitochondria (Fucho et al., 2017; Bellini et al., 2018). Lipid accumulation and low expression of CPT1α support the damaged FAO capability (Gray et al., 2013; Ghosh et al., 2017) observed in the STZ/HFD-induced mouse kidneys. Indeed, we examined the expression of the FAO rate-limiting enzyme CPT1α in HUVECs and mouse kidneys and found that it was decreased under HGHF. Besides, HGHF inhibits fatty acid utilization and increases the secretion of endothelial FN. Consistent with this, TGF-β1 and interleukin-1β can trigger endothelial-to-mesenchymal-transition by affecting acetyl-CoA levels through FAO (Xiong et al., 2018). We also found that when FAO was inhibited with or without silencing PACS2, the endothelial barrier was impaired, and Smad2 was activated. These endothelial dysfunctions caused by FAO inhibition cannot be offset by PACS2 knockdown, indicating that FAO works downstream of PACS2.

High glucose can activate endothelial nitric oxide synthase and induce mtROS that cause mitochondrial DNA damage. Our previous studies found that high glucose affects dynamin-related protein 1-mediated mitochondrial fission in endothelial cells (Liu et al., 2019a). In this study, we first observed an increase in MAMs under HGHF. Abnormally increased MAMs have been reported to evoke mitochondrial calcium overload, mitophagosome formation, and mitophagy (Gbel et al., 2020). It also has been reported that MAMs are associated with hepatocyte insulin resistance (Bassot et al., 2019) and diabetic smooth muscle cell phenotypic transformation (Moulis et al., 2019). We found that under HGHF, increased endothelial MAMs were accompanied by mitochondrial swelling and fragmentation. Thus, we explored the significance of the increase in MAMs in endothelial cells. Previous studies have shown that MAMs can regulate Ca^2+^ signalling (Boyman et al., 2020), lipid synthesis (Balla et al., 2020), and mitochondrial fusion (Hu et al., 2021). Although we assessed three enriched proteins related to lipid metabolism (Thomas et al., 2017), including GRP75, FACL4, and PACS2, only PACS2 showed significant changes after HGHF treatment. As one of the enriched regulatory proteins of MAMs, mice lacking PACS2 still maintain relatively low levels of MAMs, which may help endothelial cells maintain a baseline crosstalk between ER and mitochondria. Previous studies have shown that downregulation of PACS2 can prevent the increase of mitochondrial Ca^2+^-mediated apoptosis (Barroso-González et al., 2016). However, we believe that PACS2 is a metabolic switch, not just a single specific pathway component in endothelial cells. In metabolism-related evaluations, downregulation of PACS2 increased NADPH/NADP^+^ ratio, ATP generation, and PA-OCR in HGHF-treated endothelial cells. Although different metabolic conditions may affect endothelial cell function, in this study, the barrier function and fibroblastic phenotype related to VE-cadherin and p-Smad2 were regulated by PACS2.

At last, extrapolating data from HUVECs *in vitro* exposure to HGHF to diabetic patients is insufficient. Ideally, studies of endothelial cells from human donors with diabetes or glomerular endothelial cell line (GEnCs) with diabetes would be used to validate the key findings. Besides that, the endothelial-specific gene knock-off animal model will be the future research plan to strengthen our evidence.

## Conclusion

In summary, our findings reveal the role of PACS2 in regulating free fatty acids metabolism in glomerular endothelial cells and DN. Therefore, knocking down PACS2 can alleviate vascular barrier damage and glomerulosclerosis by enhancing the FAO ability. We highlight the metabolic mechanism and provide a new target for the treatment of diabetic microvascular complications.

## Data Availability

The datasets presented in this study can be found in online repositories. The names of the repository/repositories and accession number(s) can be found below: The protemics data has uploaded *via* PRIDE in ProteomeXchange with accession number: PXD033227.

## Supplementary Material

Supplementary Figure S1 Flow chart of STZ/HFD mouse model. A. Schematic diagram of diabetic mice model induced by streptozotocin (stz) and fed with high-fat diet (HFD) for up to 20 weeks.

Supplementary Figure S2 Proteomics of HUVECs under HGHF. **(A)**. Heap map of significantly different proteins between control and HGHF(test) group. **(B)**. Differential proteins located in mitochondria and ER **(C)**. Proteins has been reported correlated with renal function (Test/Control).

Supplementary Figure S3 Metabolomics of HUVECs under HGHF. **(A)**. PCA map of samples in metabolomics. **(B)**.Differential metabolomic molecular in HUVECs under HGHF. **(C)**. Long-chain free fatty acid correlated with FAO in metabolomic.

## Abbreviations

BUN, Blood urea nitrogen; BSA, Bovine serum albuminates; BDH1, D-beta-hydroxybutyrate dehydrogenase; CPT1α, carnitine palmitoyl-transferase 1α; CHO, Total cholesterol; CRE, Creatinine; DN, Diabetic nephropathy; DKD, Diabetic kidney disease; ESRD, End-stage renal disease; EC, Endothelial cell; ECM, Extracellular matrix; ETO, Etomoxir; FN, Fibronectin; FAO, Free fatty oxidation; GO, Gene ontology; HFD, High-fat diet; HGHF, High glucose and high fat; ITGB4, Integrin beta 4; ITGA3, Integrin alpha 3; KEGG, Kyoto Encyclopedia of Genes and Genomes; MAMs, Mitochondria-associated membranes; MLLT4, Myeloid/lymphoid or mixed-lineage leukemia 4; OCR, Oxygen consumption rate; PACS2, Phosphofurin acidic cluster sorting protein 2; PA, Palmitic acid; STZ, Streptozocin; TG, Triglyceride; TR-F, Time-resolved fluorescence; u-mALB, Urine microalbuminuria.
