# Supplementary figures and images for: AKT/PACS2 Participates in Renal Vascular Hyperpermeability by Regulating Endothelial Fatty Acid Oxidation in Diabetic Mice
Source: Front Pharmacol. 2022 Jul 5;13:876937. doi: 10.3389/fphar.2022.876937 (PMC9294407; doi:10.3389/fphar.2022.876937)

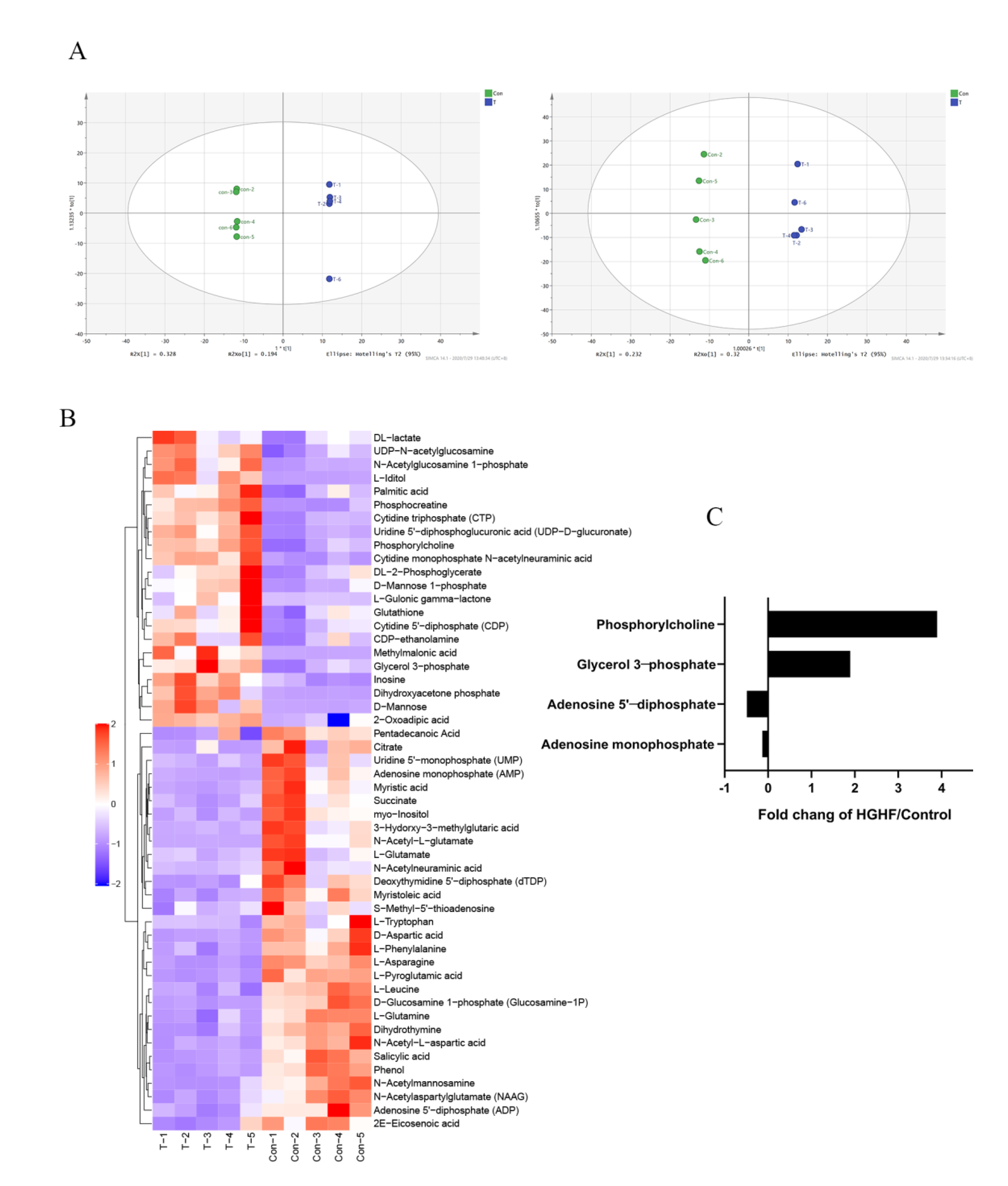

Supplement: Supplementary file 1 [file Image3.TIF]

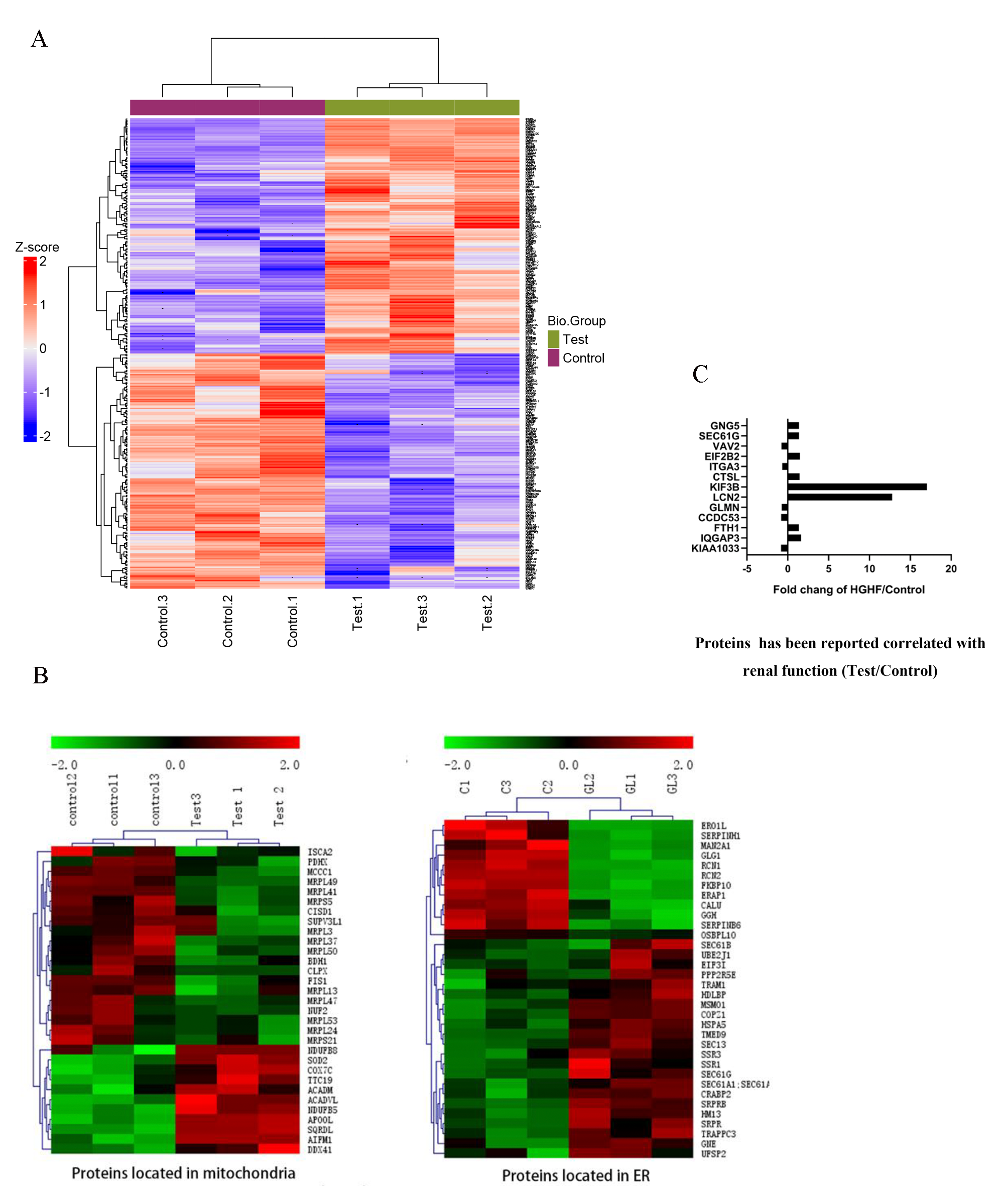

Supplement: Supplementary file 2 [file Image2.TIF]

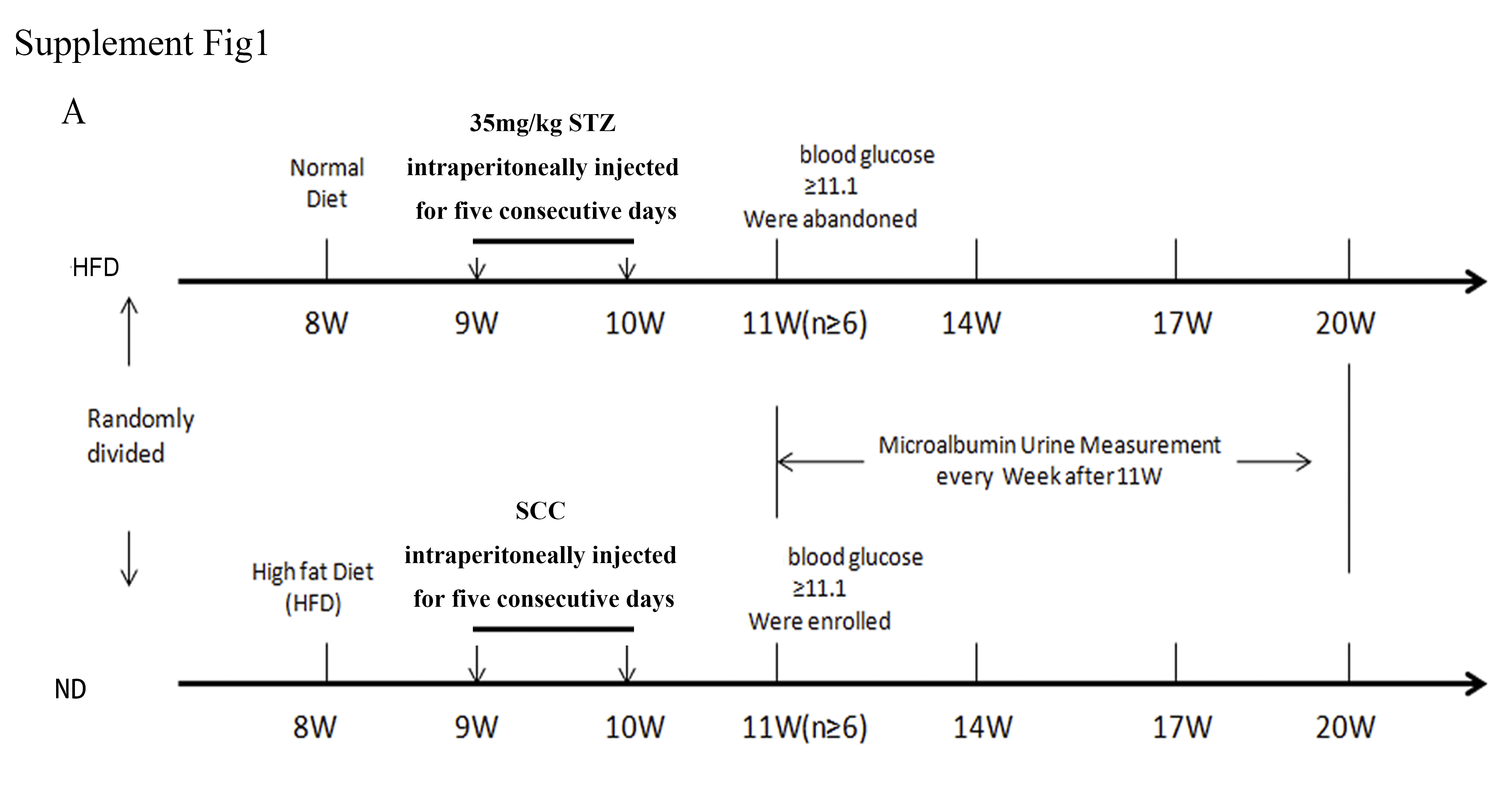

Supplement: Supplementary file 3 [file Image1.TIF]
